# Real‐Time NMR Recording of Fermentation and Lipid Metabolism Processes in Live Microalgae Cells

**DOI:** 10.1002/anie.202117521

**Published:** 2022-02-15

**Authors:** Faezeh Nami, Maria Joao Ferraz, Thomas Bakkum, Johannes M. F. G. Aerts, Anjali Pandit

**Affiliations:** ^1^ Dept. of Solid-State NMR Leiden Institute of Chemistry Leiden University Einsteinweg 55 2333 CC Leiden The Netherlands; ^2^ Dept. of Medicinal Biochemistry Leiden Institute of Chemistry Leiden University Einsteinweg 55 2333 CC Leiden The Netherlands; ^3^ Dept. of Bio Organic Synthesis Leiden Institute of Chemistry Leiden University Einsteinweg 55 2333 CC Leiden The Netherlands

**Keywords:** Biophysics, *Chlamydomonas*, Glycolipids, Live-Cell NMR, Magic-Angle Spinning

## Abstract

Non‐invasive and real‐time recording of processes in living cells has been limited to detection of small cellular components such as soluble proteins and metabolites. Here we report a multiphase NMR approach using magic‐angle spinning NMR to synchronously follow microbial processes of fermentation, lipid metabolism and structural dynamic changes in live microalgae cells. Chlamydomonas reinhardtii green algae were highly concentrated, introducing dark fermentation and anoxia conditions. Single‐pulse NMR experiments were applied to obtain temperature‐dependent kinetic profiles of the formed fermentation products. Through dynamics‐based spectral editing NMR, simultaneous conversion of galactolipids into TAG and free fatty acids was observed and rapid loss of rigid lipid structures. This suggests that lipolysis under dark and anoxia conditions finally results in the breakdown of cell and organelle membranes, which could be beneficial for recovery of intracellular microbial useful products.

## Graphical abstract

The application of solid‐state MAS NMR on live microalgae for monitoring of fermentation, lipid metabolism and structural dynamics changes is demonstrated. Conversion of galactolipids into TAG and free fatty acids is observed, accompanied with rapid loss of rigid lipid structures. It is concluded that high cell densities, creating dark, anoxia conditions, induce lipid catabolism and cell death resulting in breakdown of cell and organelle membranes.

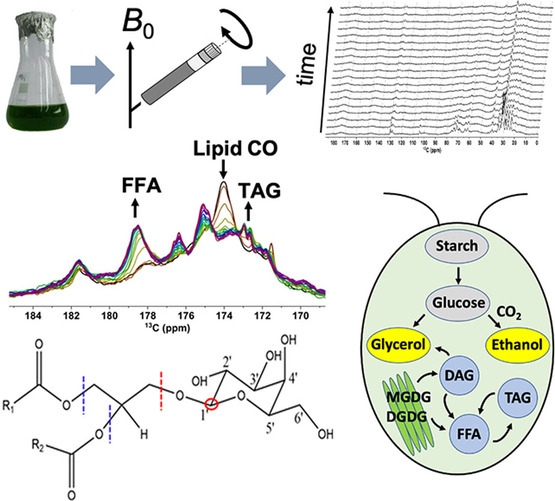

Real‐time monitoring of biological events in living cells has long‐been recognized for its importance for microbial cell factories, drug discovery and identification of disease markers. In this regard, NMR spectroscopy gained more momentum during the last two decades[^1^, ^2^, ^3^] owing to technological developments and is the method of choice as a non‐invasive technique, which allows the real‐time detection of multiple target compounds at their endogenous level of expression in the cellular environment. Unlike fluorescence‐based techniques, which require chemically attached probes, NMR spectroscopy can be applied on the endogenous cell compounds.[^4^, ^5^, ^6^, ^7^] In recent years, real‐time NMR approaches have been successfully applied to both prokaryotes and eukaryotic live cells to study cell metabolism,[^8^, ^9^, ^10^, ^11^] protein folding and maturation,^[12]^ redox reactions,[^13^, ^14^] and ligand/metal binding.^[15]^ Multiphase NMR spectroscopy has been designed to differentiate liquid‐, gel‐ and solid‐like components in complex mixtures such as soil and algae biomass extractions[^16^, ^17^] Furthermore, solution‐state NMR and ultraslow MAS NMR and high‐resolution (HR) MAS NMR have been applied to determine the availability of aquatic carbon for green algae and metabolites inside living water fleas, fungus, and nematodes.[^18^, ^19^, ^20^, ^21^] However, live‐cell NMR applications have been limited to detection of soluble cellular components such as small molecules and soluble proteins.

Herein, we describe a real‐time multi‐phase magic‐angle spinning (MAS) NMR approach to simultaneously monitor cellular processes and structural dynamics changes, and show that we can synchronously study fermentation, structural dynamics and lipid metabolism in live microalgae cells. Microalgae are considered to have high potential for production of food, pharmaceuticals and biofuels as renewable energy sources.^[22]^ In a closed experimental system, lack of light, oxygen and high cell density triggers fermentation and eventually may lead to autolysis, which can be beneficial for extraction of valuable compounds.^[23]^ In this work, we subjected cells of the unicellular green algae *Chlamydomonas reinhardtii* (*Cr*.) to dark, anoxia conditions that trigger fermentation, by concentrating the cell suspensions in MAS NMR rotors, and subsequently followed time‐dependent in‐cell changes by solid‐state NMR spectroscopy.

To investigate the effect of high cell density and MAS on cell survival, the viability of *Cr*. cells concentrated to an OD_750_ of ≈120 into a 4 mm MAS NMR rotor by mild centrifugation was assessed by flow cytometry. Flow cytometry was performed on static rotor samples and on samples that were held at MAS NMR conditions by 5 kHz spinning under a magic angle inside a 17.4 T magnet. Fluorescein diacetate (FDA) was used as a viability probe, which measures a combination of enzymatic activity and cell‐membrane integrity. FDA is a non‐fluorescent molecule and is hydrolysed by esterase enzymes to fluorescent fluorescein in live cells. Fluorescein is only retained by cells with intact cell membranes, while damaged cells quickly lose the fluorophore (even if some residual esterase activity remains) and become non‐fluorescent, which are then considered as non‐viable cells. The representative flow cytometric graphs of *Cr*. cells stained with FDA are shown in Figure S1, S2. The viability of cells equilibrated at 23 °C after 3.5 hours of MAS at 5 kHz inside the 17.4 T magnet was found to be 55 % with respect to fresh cell cultures and further dropped to 4 % after 6.5 hours of MAS (Figure 1).

**Figure 1:**
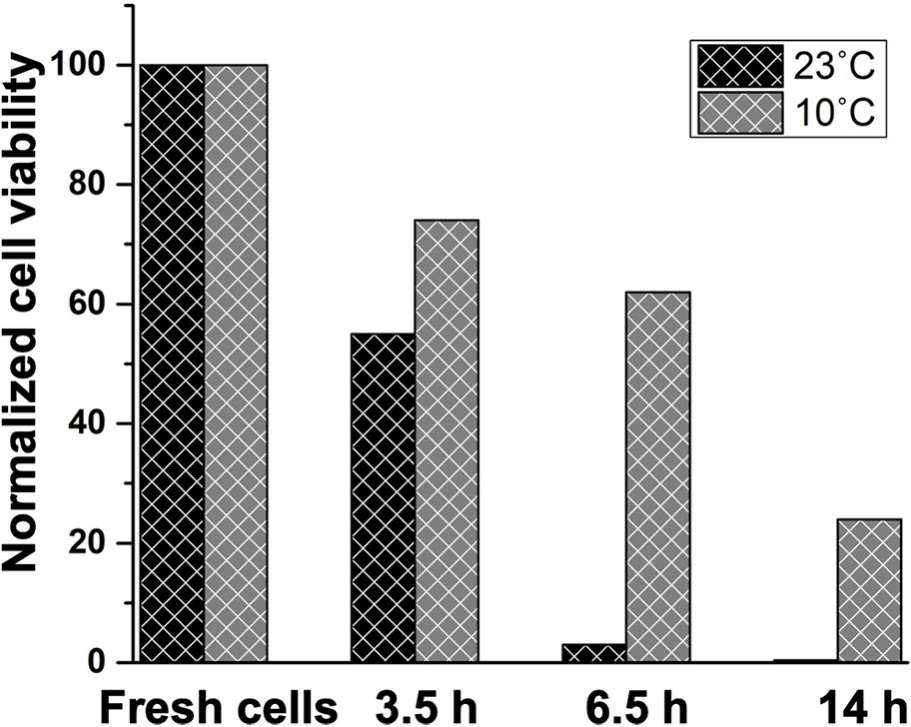
Flow cytometry‐based cell viability of *Cr*. cells kept in 4 mm rotors at MAS NMR conditions. Cells were subjected to 5 kHz MAS at 10 °C or 23 °C for the duration of time indicated on the *x*‐axis, after which the viability assays with FDA were performed. The FDA fluorescence of fresh *Cr*. cells is normalized at 100 % viability.

Lowering the temperature to 10 °C resulted in prolongation of cell survival to 74 % and 62 % after 3.5 and 6.5 hours of MAS at 5 kHz, respectively. To investigate the effect of spinning at 5 kHz on the cell viability, cell suspensions were also rotor‐incubated at similar high densities without MAS and kept outside the magnet. Figure S3 shows comparable cell viability of those static samples over time, dropping to 5 % after 6.5 hrs at 23 °C. In contrast, 75 % cell viability was observed after 6.5 hours for diluted cell suspensions of OD_750_≈17. We conclude therefore that the *Cr* cell survival rates depended on the cell density and temperature of incubation, and were not affected by spinning at 5 kHz MAS.

For MAS NMR experiments, uniformly ^13^C labelled *Cr*. cells were packed into a 4 mm MAS NMR rotor using mild centrifugation and repeated series of three types of NMR pulse experiments were performed at 23 °C or at 10 °C equilibration temperature, over a time span of 24 hours. We first discuss the results of ^13^C MAS NMR‐based direct polarization (DP) single‐pulse experiments, in which the signals of all 13‐carbon constituents are detected. The DP spectra of *Cr*. cells after 1 hr and 24 hr series of MAS NMR experiments at 23 °C are presented in Figure 2. In these spectra, sharp peaks between 10–40 ppm involve the signals of CH_3_ and CH_2_ carbons of metabolites and lipid acyl chains, which are superimposed on the broad band of accumulated protein side‐chain^13^C signals.[^23^, ^24^, ^25^, ^26^, ^27^, ^28^] Signals of carbons with an OH functional group and of the protein backbone C_α_ accumulate in the region between 50–70 ppm. In the region between 70–110 ppm, signals of carbohydrate contents accumulate, including signals of the cell‐wall hydroxyproline‐rich glycoproteins and the head groups of galactosyl lipids, which are the most abundant type of lipids in *Cr*. cells.[^24^, ^25^, ^26^, ^27^, ^28^, ^29^, ^30^] The signals of protein aromatic side‐chains and double‐bonded ^13^C signals of the unsaturated lipid chains are visible in the region between 125–135 ppm. The carbonyl and carboxyl signals from lipids and protein backbone appear between 170–180 ppm.[^24^, ^25^, ^26^, ^27^, ^28^, ^29^, ^30^] A comparison of the initial DP spectrum and the DP spectrum after 24 hours shows the formation of metabolic end‐ and by‐products over time as indicated in Figure 2. We identify signals that emerge in time of ethanol (17.4 ppm and 58.1 ppm), glycerol (63.2 ppm and 72.6 ppm) and aqueous CO_2_ (125.1 ppm).^[25]^ Those are fermentation products that have been reported for *Cr*. under dark, anaerobic conditions. Similarly, signals at 76.5 ppm, 92.7 ppm and 96.5 ppm develop in time that were previously attributed to starch by‐products^[31]^ and potentially originate from a‐ and β‐glucose. The same products are detected in the NMR spectra of *Cr*. cells equilibrated at 10 °C (Figure S5). Ethanol and glycerol are also detected in solution NMR spectra that were collected from the washed‐out cell supernatant (Figure S4), indicating that these products are exerted from the cells.

**Figure 2:**
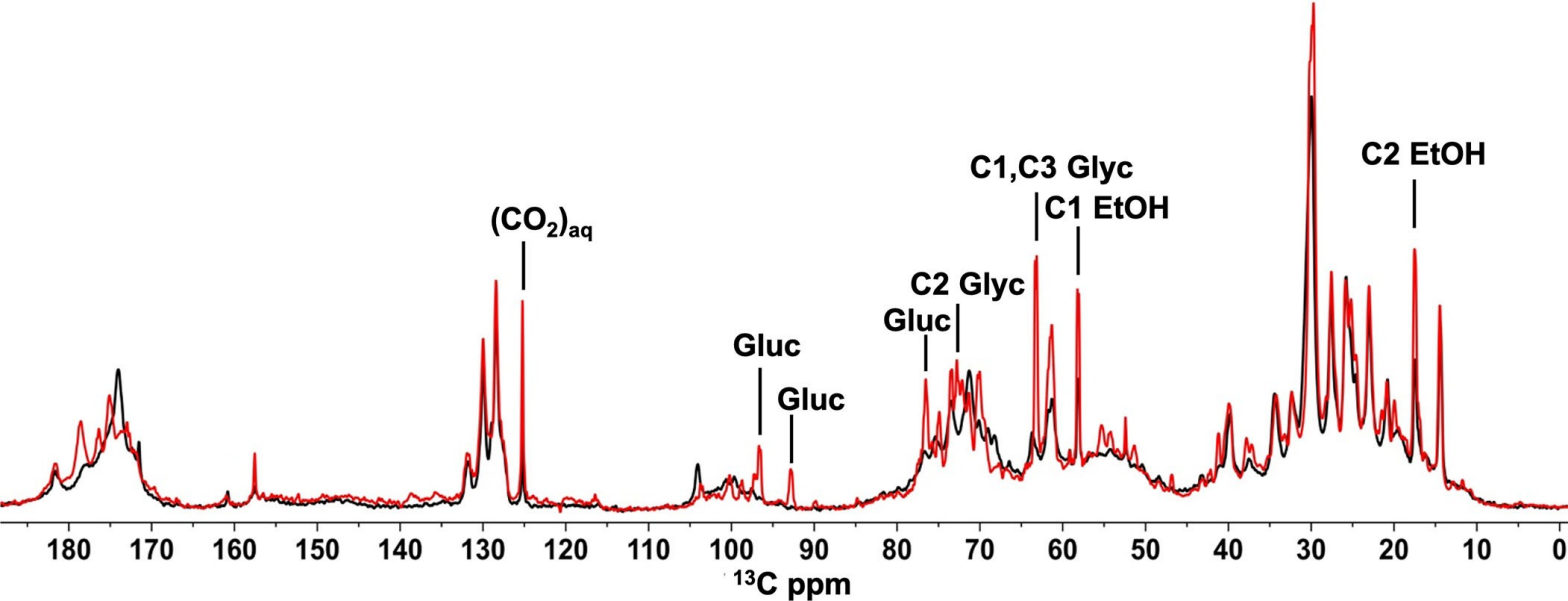
Overlaid DP ^13^C NMR spectra of *Cr*. cells after 1 h (black) and 24 h (red) of 5 kHz MAS at 23 °C. Signals of ethanol (EtOH), glycerol (Glyc), glucose (Gluc) and aqueous CO_2_ are indicated.

To gain insight in the kinetic profiles of the fermentation products, we analysed the series of DP spectra that were recorded with intervals of 77 minutes. The time‐dependent DP spectra at 23 °C are shown in Figure 3A, zooming in on the carbohydrate region, where notable spectral changes are observed. The full spectra are presented in Figure S6 and S7. Kinetic plots of the NMR signal intensities of ethanol, glycerol and CO_2_ at 23 °C and at 10 °C are shown in Figure 3B–D, respectively. All curves are fitted with a first‐order exponential function. Ethanol and CO_2_ signals follow the same pattern of steep initial increase and reach a plateau after ≈5 hours at 23 °C and after ≈9 hours at 10 °C, indicating that the cells remain metabolically active for longer time at the lower temperature in line with the observed prolonged cell viability. The slight decrease of ethanol signal at longer times could be due to evaporation from the NMR rotor.

**Figure 3:**
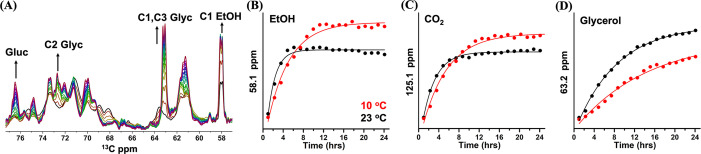
A) Time‐dependent series of DP ^13^C ssNMR spectra of *Cr*. cells at 23 °C, zoomed‐in region of ethanol (EtOH), glycerol and carbohydrate (see arrows) followed for 24 hours with the time interval of 77 minutes. B)–D) DP NMR signal intensities at 10 °C (red) and 23 °C (black) as a function of time for ethanol (58.1 ppm, B), CO_2_ (125.1 ppm, C) and glycerol (63.2 ppm, D). For visual comparison of the kinetic profiles, the intensities of spectra collected at 23 °C and at 10 °C are normalized at the first time point.

Interestingly, the rates for production of both compounds, which are end products of starch fermentation in *Cr*. are similar, as shown in Table S1. Unlike ethanol and CO_2_, production of glycerol continues after 5–10 hours, and consequently the total produced amount of glycerol is higher for the cells equilibrated at 23 °C than at 10 °C, while the total amount of produced ethanol is higher for cells kept at 10 °C.

In addition to fermentation, subsequent lipolysis and formation of TAG is observed. The galactolipid composition of *Cr*. cells consists of monogalactosyldiacylglycerol (MGDG), and digalactosyldiacylglycerol (DGDG) in descending order of abundance.[^32^, ^33^, ^34^] Galactolipids form the major lipid constituents of *Cr*. chloroplast thylakoid membranes. Lipid metabolism, which plays a pivotal role in the survival of the *Cr*. cells involves sequential release of head groups from diacylglycerol (DAG) followed by release of free fatty acid (FFA) from this lipid that is subsequently metabolized to acetyl‐CoA via β‐oxidation or used to form triacylglycerol (TAG).^[34]^ Furthermore, considering that we observe the loss of cell viability in time, at later stages lipid hydrolysis by unspecific esterases might occur in the fractions of death cells. These steps are visible in our NMR experiments and supported by high‐performance thin‐layer chromatography (HPTLC, Figure S8). The C1′ carbons of MGDG and DGDG galactosyl headgroups have a distinct NMR signature (Figure S9), and appear as a merged peak at 104 ppm in the *Cr*. DP MAS NMR spectrum, while the C1′′ carbon of the second galactose ring in the DGDG headgroup is identified at 99 ppm (Figure S9, S10 and S11). The same signals are also identified in spectra of cell wall‐deficient mutant cells, confirming their attribution to lipid and not to glycoproteins of the cell wall (Figure S11). FFA contain carboxylic acids of which the carbons have downshifted NMR resonance signals compared to the lipid carbonyls and resonate between 178–179 ppm. TAG and DAG have distinct 13‐carbon NMR signals resonating between 173.1–173.4 (DAG) and 172.4–172.9 ppm (TAG).[^35^, ^36^] Figure 4A shows time‐dependent DP NMR spectra of cells collected at 23 °C, focussing on the region between 170–180 ppm. Clearly, a trend is observed where a decrease of signal centered at 174 ppm, assigned to the lipid carbonyls, (Figure S12, Supporting Information section) is accompanied with the rise of a band at 178.5 ppm that we attribute to FFA. Additional small bands emerge at 172.9 and 172.4 ppm that we tentatively attribute to TAG (see arrow in Figure 4A and difference spectra in Figure S11B). Kinetic plots of the DP NMR signal intensities of MGDG, lipid carbonyl and FFA are presented in Figure 4B and C and show a sigmoidal pattern where the decay of lipid galactosyl signal mirrors the rise of the FFA signal. Lipid metabolism was further confirmed by HPTLC that unambiguously shows the decrease of MGDG and DGDG and the increase of FFA and TAG as a function of cell incubation time (see Figure S8). Notably, the final concentration of FFA, which is proportional to the integrated DP NMR FFA signal intensity, is higher than that of TAG, as confirmed by the HPTLC data.

**Figure 4:**
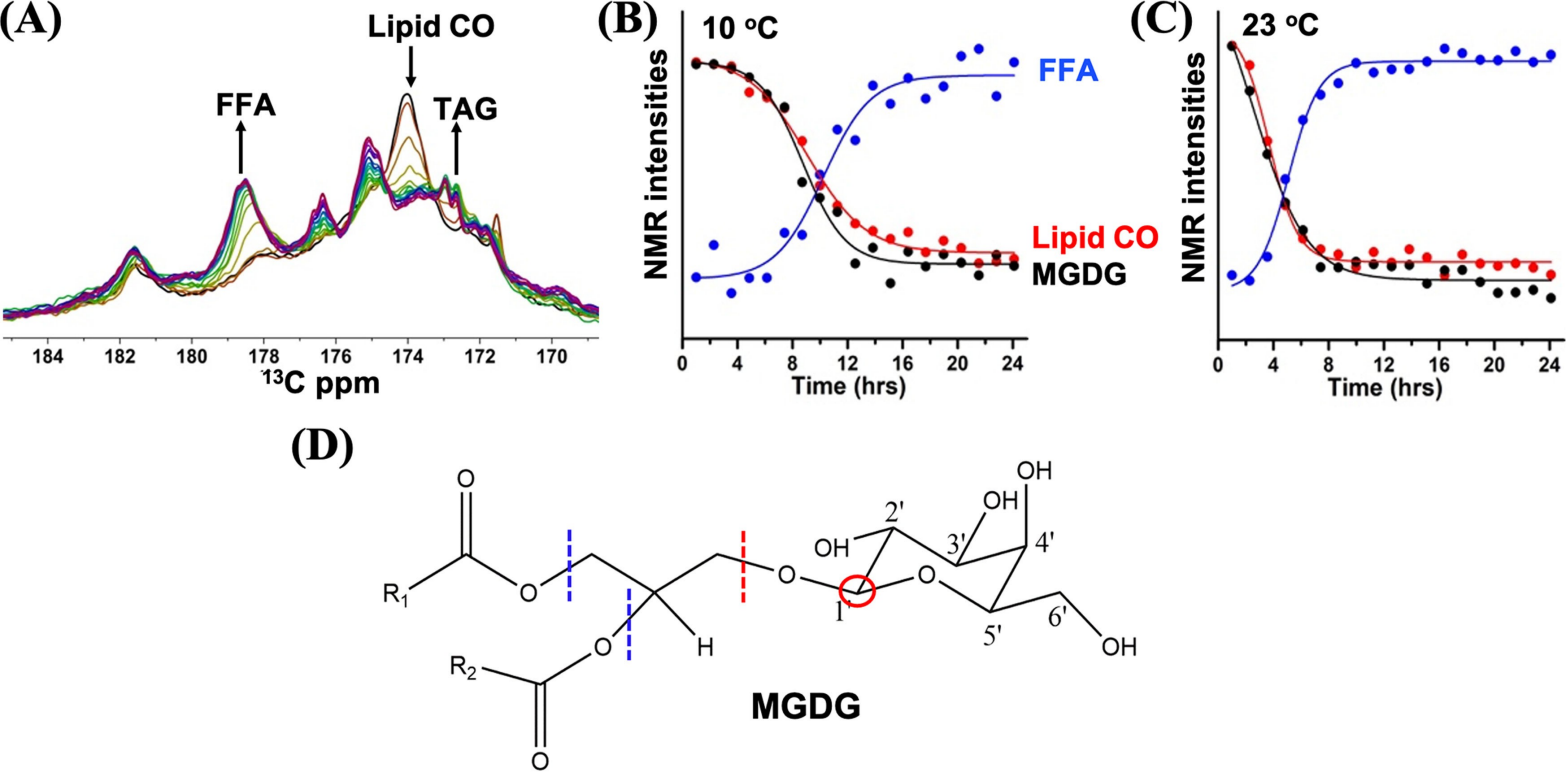
A) Time‐dependent series of DP ^13^C NMR spectra of *Cr*. cells at 23 °C, zoomed in at the carbonyl region. Spectra were recorded over a time span of 24 hours with intervals of 77 minutes. Arrows indicate the decrease of galactolipid carbonyl signal (174 ppm) and increase of FFA and TAG. B and C) DP NMR signal intensities at 10 °C (B) and 23 °C (C) of MGDG galactosyl (104 ppm, black), lipid carbonyl (174 ppm, red) and FFA (178.5 ppm, blue), showing increase of FFA and loss of galactolipid in time. For visual comparison, the NMR signal intensities at 104 ppm and 174 ppm are normalized at the first time point, while the NMR signal intensities at 178.5 ppm are normalized at *t*=24 hours. D) Schematic drawing of MGDG structure indicating C1′ (red circle) and sites of cleavage to release the headgroup (red dashed line) and FFA (blue dashed lines).

To shed more light on structural dynamics changes, we applied MAS NMR dynamic‐based spectral editing cross polarization (CP) and insensitive nucleus‐enhanced polarization‐transfer (INEPT) experiments. CP and INEPT are selective for detection of rigid (macro)structures with micro‐ to millisecond dynamics and of dynamic structures, including small molecules, with (sub)nanosecond dynamics, respectively. Time‐dependent CP spectra of cells equilibrated at 23 °C are shown in Figure 5A, zooming in on the aliphatic and carbohydrate region. The full CP and INEPT spectra of cells equilibrated at 23 °C and 10 °C are presented in Figure S13–S20, Supporting Information section.

**Figure 5:**
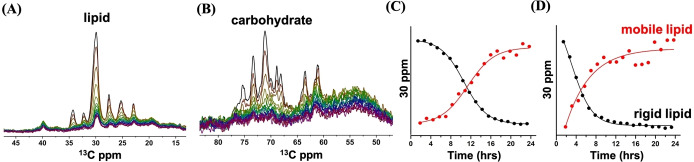
A) Time‐dependent series of CP ^13^C ssNMR spectra of *Cr*. cells at 23 °C, zoomed‐in aliphatic (15–45 ppm) and B) carbohydrate (50–80 ppm) region. Spectra were recorded over a time span of 24 hours with intervals of 77 minutes and decay in time. C and D) CP (black) and INEPT (red) NMR lipid signal intensities (30 ppm) at 10 °C (B) and 23 °C (C), showing increase of mobile (INEPT) and loss of rigid (CP) lipid structures in time. For visual comparison, CP and INEPT signal intensities were normalized at the first time point and at t=24 hours, respectively.

In contrast to the DP NMR spectra that are insensitive to changes in structural dynamics, the CP spectra show a rapid loss of the sharp peaks that are representative of lipid and carbohydrate signals (Figure 5A and B), implicating a major loss of rigidity or breakdown of lipid membranes and carbohydrate macrostructures. The INEPT spectra that detect dynamic (small) molecules show a parallel increase of lipid signal, and signals emerging at 76.5 ppm, 92.7 ppm and 96.5 ppm (Figure S15–S18) of glucose products, consistent with the breakdown of carbohydrate structures and increase of lipid fluidity and/or loss of membrane structures. Figure 5C and D plot the loss of rigid, CP (black) and build‐up of mobile, INEPT (red) lipid NMR intensities over time. The similar kinetic profiles of lipid dynamic changes, loss of galactolipid and accumulation of TAG and FFA suggests that hydrolysis of galactolipids is associated with disruption of (galacto)lipid membranes. The loss of carbohydrate signal in the CP spectra further points to the degradation of starch, consistent with the build‐up of glucose products and accumulation of glycerol. Each series of NMR experiments were performed at least two times and the data reproducibility are shown in Figure S21 and S22.

In summary, we introduced a live cell MAS NMR approach that enables the simultaneous monitoring of cellular metabolic, catabolic and structural‐dynamic changes upon dark concentration of *Cr*. microalgae in a real‐time manner as depicted in Figure 6, enabling us to correlate processes based on their kinetic profiles. Microbial *Cr*. cell suspensions incubated at high cell densities switch to fermentation metabolism, producing ethanol and glycerol, and subsequently catabolize galactolipids to TAG and FFA. The similar kinetics of ethanol and CO_2_ product formation indicates that the last step in the fermentation process towards ethanol production from acetaldehyde or via acetyl‐CoA, which does not release CO_2_, is not the rate‐limiting step.

**Figure 6:**
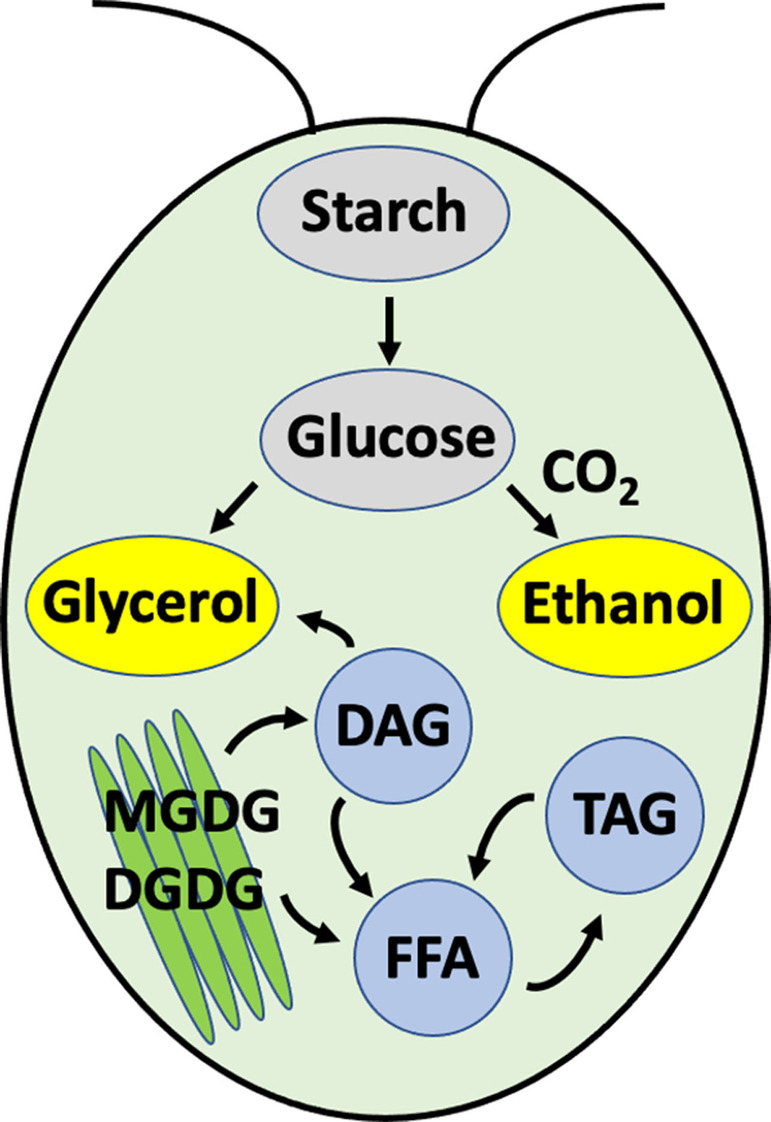
Schematic depiction of the cellular processes that are simultaneously observed by MAS NMR on living *Cr*. algae at high cell densities.

In this study MAS spinning at 5 kHz does not appear to be the dominant factor that leads to cell death. As a result of the high cell densities used to stimulate dark fermentation/anoxia conditions, cell death occurs within 5–9 hours, depending on the incubation temperature, correlating with plateauing of ethanol and CO_2_ production. Future work should look at providing air exchange via a drilled rotor cap that has been shown to extend the life span of multicellular living organism.^[37]^ In parallel with the fermentation processes and accumulation of FFAs, significant breakdown of rigid lipid structures is observed. This could be the result of lipid metabolism, and at later time points of lipid hydrolysis by esterases occurring in dead cells. Remarkably, glycerol production was observed from the start of the experiments and continued for 24 hours after rotor incubation, exceeding the cell survival times. This suggests that glycerol is produced as fermentation product and further accumulates via other reactions occurring in the cell lysates. Lowering of the incubation temperature slows down the rates of product formation but could be beneficial to prolong cell viability.^[38]^ The heating impact of high‐power decoupling (80 kHz) during the CP experiments would need to be investigated further in living algae samples, while it was shown that 50 kHz is fine even for dry algae.^[38]^ The product total yields are differently affected by the set temperature, as lowering of the incubation temperature from 23 °C to 10 °C reduced the yield of glycerol production but resulted in increased ethanol product yield.

Finally, the observed breakdown of lipid membranes as a result of dark anoxia‐induced processes may facilitate extraction of intracellular useful products from the cells.

## Conflict of interest

The authors declare no conflict of interest.

## Supporting information

As a service to our authors and readers, this journal provides supporting information supplied by the authors. Such materials are peer reviewed and may be re‐organized for online delivery, but are not copy‐edited or typeset. Technical support issues arising from supporting information (other than missing files) should be addressed to the authors.

Supporting Information

## Data Availability

The data that support the findings of this study are available from the corresponding author upon reasonable request.
