# Supplementary material for: Real‐Time NMR Recording of Fermentation and Lipid Metabolism Processes in Live Microalgae Cells
Source: Angew Chem Int Ed Engl. 2022 Feb 15;61(14):e202117521. doi: 10.1002/anie.202117521 (PMC9305762; doi:10.1002/anie.202117521)
Supplement: Supplementary file 1 — Supporting Information [file ANIE-61-0-s001.pdf]

Angewandte Chemie
Eine Zeitschrift der Gesellschaft Deutscher Chemiker

Supporting Information

Real-Time NMR Recording of Fermentation and Lipid Metabolism Processes in Live Microalgae Cells

*F. Nami, M. J. Ferraz, T. Bakkum, J. M. F. G. Aerts, A. Pandit**

SUPPORTING INFORMATION

Table of Contents

1. Experimental Procedures

1.1 Cell culturing ----------3
1.2 Sample preparation for solid-state NMR ----------3
1.3 Solid-state NMR experiments ----------3
1.4 Solution NMR experiments ----------3
1.5 Cell viability ----------3
1.6 Flow cytometry experiments ----------3
1.7 Lipid isolation ----------4
1.8 High-performance thin-layer chromatography ----------4
1.9 ^{13}C NMR assignments of monogalactosyldiacylglycerol (MGDG), digalactosyldiacylglycerol (DGDG), lipid carbonyl and triacylglycerol (TAG) ----------4

2. Supplementary figures

S1 Representative flow cytometric graphs showing the gating strategy ----------6
S2 Flow cytometric fluorescence histograms for *Cr.* cells after ssNMR experiments ----------7
S3. Flow cytometric fluorescence histograms comparing incubated *Cr.* cells with and without MAS ----------8
S4 Solution NMR spectrum of washed-out cells supernatant after MAS ssNMR ----------9
S5 Overlaid DP ^{13}C ssNMR spectra of *Cr.* cells after 1 h and 24 h of MAS ----------10
S6 Stacked time-dependent DP ^{13}C ssNMR spectra of *Cr.* cells at 23 ˚C ----------11
S7 Stacked time-dependent DP ^{13}C ssNMR spectra of *Cr.* cells at 10 ˚C ----------12
S8 HPTLC analysis of total lipids content of *Cr.* cells - ----------13
S9 Molecular structure of MGDG and solution NMR spectra of MGDG and DGDG ----------14
S10 ^{13}C-^{13}C INEPT-TOBSY and ^{1}H-^{13}C HETCOR spectrum of thylakoid membranes ----------15
S11 Overlaid DP ^{13}C ssNMR spectrum of cell wall-deficient *Cr.* cells and *Cr.* cells after 1h and 24h----------16
S12 Overlaid DP ^{13}C ssNMR spectrum of thylakoid membranes and *Cr.* cells after 1h and 24h----------17
S13 Overlaid CP ^{13}C ssNMR spectra of *Cr.* cells after 1 h and 24 h of MAS at 23 ˚C ----------18
S14 Stacked time-dependent CP ^{13}C ssNMR spectra of *Cr.* cells at 23 ˚C ----------19
S15 Overlaid CP ^{13}C ssNMR spectra of *Cr.* cells after 1 h and 24 h of MAS at 10 ˚C ----------20
S16 Stacked time-dependent CP ^{13}C ssNMR spectra of *Cr.* cells at 10 ˚C ----------21
S17 Overlaid INEPT ^{13}C ssNMR spectra of *Cr.* cells after 1 h and 24 h of MAS at 23 ˚C ----------22
S18 Stacked time-dependent INEPT ^{13}C ssNMR spectra of *Cr.* cells at 23 ˚C ----------23
S19 Overlaid INEPT ^{13}C ssNMR spectra of *Cr.* cells after 1 h and 24 h of MAS at 10 ˚C ----------24
S20 Stacked time-dependent INEPT ^{13}C ssNMR spectra of *Cr.* cells at 10 ˚C ----------25
S21 Reproducibility of DP NMR signal intensities at 23 ˚C and 10 ˚C ----------26
S22 Reproducibility of CP and INEPT NMR signal intensities at 23 ˚C and 10 ˚C ----------27

3. Supplementary table

ST1 The rate constants of metabolites formed during MAS NMR experiments of *Cr.* cells ----------28

4. References ----------29

1. Experimental Procedures

1.1 Cell culturing

Wild-type *Chlamydomonas reinhardtii* (*Cr.*) cells (strain cc124) and cell wall-deficient mutant (cw15) were grown mixotrophically in tris-acetate-phosphate (TAP) medium at pH 7 in a home-built set up, under continuous illumination with white LEDs (~50 µmol/m^2 s) and constant temperature of 25 °C. For ^{13}C isotope-label incorporation for NMR experiments, the acetic acid in the TAP medium was replaced by ^{13}C acetic acid (Cambridge Isotopes, Massachusetts, USA).

1.2 Sample preparation for solid-state NMR

The *Cr.* cells were harvested at exponential phase using centrifugation and washed with high-salt medium[1] to avoid the presence of ^{13}C acetic acid during NMR experiments. The cells were then spun down into a 4 mm NMR rotor by mild centrifugation (~1000 x g).

1.3 Solid-state NMR experiments

Solid-state NMR (ssNMR) experiments were performed on a Bruker Avance-I 750 MHz wide bore spectrometer using 4 mm triple-resonance (^{1}H, ^{13}C, and ^{15}N) magic-angle-spinning (MAS) probe head. All spectra were recorded at a spinning MAS frequency of 5 kHz. Direct polarization (DP) spectra were recorded with 6 s recycle delay and 22 ms acquisition time. For cross- polarization (CP) a contact time of 2 ms, recycle delay of 6 s and acquisition time of 22 ms, ω1C/2π of 40.3 kHz and ^{1}H nutation frequency linearly ramped from 80 to 100 kHz were used. The RF field for high-power decoupling was set at 80 kHz. Insensitive nucleus- enhanced polarization-transfer (INEPT) spectra were recorded with two delays of 1.25 ms and an acquisition time of 40 ms. The MAS ssNMR data were processed and analyzed in Top-Spin 4.1.1 and MestReNova 12.0.1.

1.4 Solution NMR experiments

After MAS ssNMR experiments, the cells from NMR rotor were washed two times with phosphate-buffered saline (PBS) buffer to collect the metabolites exerted from the cells. The washed-out cell supernatant were mixed with 10% D_2O (final concentration) to enable field locking.

Solution NMR experiments were performed on a Bruker Avance-I 500 MHz spectrometer using 5 mm BBFO Z-gradient high resolution probe head.

1.5 Cell viability

To assess cell viability after NMR experiments, cells were resuspended in PBS to an OD_{750} of 0.9 (~ 1.8 × 10^7 cells/ml) and then 30 times diluted in PBS buffer containing 5 mM ethylenediaminetetraacetic acid (EDTA) to avoid clumping of the cells during flow cytometric experiments. Cell suspensions (~ 6 × 10^5 cells/ml) were subsequently incubated with fluorescein diacetate (FDA) at a final concentration of 2.5 µM for 20 min at room temperature in dark. Two samples were used as negative controls, heat-killed cells (95˚C for 10 min to inactivate esterase activity) and cells from fresh culture without FDA. Fresh cells as a positive control were first washed and resuspend in HSM medium to an OD_{750} of 0.9 and incubated in dark before staining with FDA in PBS buffer.

1.6 Flow cytometry experiments

The flow cytometry experiments were performed on a Guava® easyCyte 12 HT Sampling Flow Cytometer (Luminex Corporation) and data analysis was performed with FlowJo V10 (FlowJo, LLC). Cells were gated on size (FSC-H vs SSC-H; ‘cells’) and shape (FSC-H vs FSC-A; ‘singlets’) to select for single cells and exclude particulate contaminations in the sample (Figure S1). FDA green fluorescence was recorded in the GRN-B channel (488 nm excitation, 520/35 nm emission) plotted on a logarithmic scale. For each measurement, at least 4000 gated cells were analysed. A stringent threshold was set to quantify only highly fluorescent cells as ‘live cells’ to minimize false positives. This threshold was set to exclude both fluorescein-negative cells (Figure S2A) and heat-killed cells (Figure S2B) from the live cell percentage. The percentage of fluorescein-positive cells was quantified for all samples and normalized on the positive control (Figure S2C), to follow the viability of the cells during NMR experiments (see Figure 1).

1.7 Lipid isolation

For total lipid extraction, the *Cr.* cells incubated under high-cell cell density (OD_{750} ≈ 120), dark and anoxia condition at 23 ˚C for 0, 2.5 and 24 hours were freeze-dried and stored at -80 ˚C until use.

The lipids of freeze-dried *Cr.* cells were extracted by Bligh and Dyer[2] in chloroform/methanol/H_2O (1:1:0.9, v/v/v). The chloroform phase was dried under nitrogen stream and the lipids were resuspended in chloroform/methanol (1:2, v/v).

1.8 High-performance thin-layer chromatography

Total lipids were analysed by one dimensional high-performance thin-layer chromatography **(**HPTLC) using Silica Gel 60 plates (10x20 cm, 0.25mm thickness, Merck, Darmstadt, Germany). For detection galactolipids and acylglycerol, the lipids were separated in the solvent system of chloroform/methanol/H_2O (65:35:2, v/v/v) and hexane/diethyl ether/acetic acid (70:30:1, v/v/v), respectively[3]. The lipids were sprayed uniformly with 8% (w/v) H_3PO_4 containing 10% (w/v) copper (II) sulphate pentahydrate and charred at 180 ˚C for 15 min[4]. Identification was achieved by co-migrating lipid standards.

1.9 ^{13}C NMR assignments of monogalactosyldiacylglycerol (MGDG), digalactosyldiacylglycerol (DGDG), lipid carbonyl and triacylglycerol (TAG)

In *Cr.* algae, the occurring lipids are (in order of abundance) MGDG, DGDG, sulfoquinovosyl-diacylglycerol (SQDG), phosphatidylglycerol (PG), phosphatidylethanolamine (PE), phosphatidylserine (PS) and Diacylglyceryl-*N,N,N* trimethylhomoserine (DGTS) and phosphatidylinositol (PI) [5]. Chloroplast thylakoid membranes contain the first four types of lipids, while PE, PS, DGTS and PI are found in the cellular membranes.

MGDG and DGDG

For assignment of MGDG or DGDG C1' and DGDG C1'' 13-carbon NMR signals of the galactosyl headgroups, we analyzed 2D spectra of *Cr*. extracted thylakoid membranes, in which the number of carbohydrate components are less redundant than in whole cells. Thylakoids were extracted from the algae according to [6]. Assignment was done using *J*-coupling based 2D ^{13}C-^{13}C INEPT-TOBSY and ^{1}H-^{13}C HetCor spectra (Fig. S10B, C) that were recorded at -3 ºC (TOBSY mixing time of 6 ms, 14 kHz MAS). A comparison with a DP MAS spectrum of cell-wall deficient *Cr*. cells (Fig. S11A, blue) further confirms that the galactosyl signals assigned to galactolipids in the spectrum of *Cr.* cells are not from glycoproteins in the cell wall. In time, the signals of MGDG and DGDG C1‘ and C1‘‘ vanish and new peaks arise at ~92 and 97 ppm that are attributed to glucose products (see Fig. S11A and B).

Lipid carbonyl

To identify the nature of the peak at 174.2 ppm, we considered the DP and CP MAS spectrum of thylakoid membranes, collected at 17 ºC, and compared this to the DP MAS spectrum of *Cr.* cells collected at 23 ºC (Fig. S12). Thylakoid membranes are densely packed with membrane-spanning proteins that make up ~75% of the total surface area [7]. The major contribution to the thylakoid spectrum in the region 170-180 ppm comes from the carbonyl signals of the protein backbones that form a broad band centered at 175 ppm [8] and are visible with DP and CP [6] as demonstrated in Fig. S12, red spectrum. Signals of the protein carboxyl side-chains (glutamate and aspartate residues) typically fall in the range 177-182 ppm depending on their protonation states [8] and are seen as peaks super-imposed on the protein CO band in the thylakoid DP spectrum. Lipid carbonyls have ^{13}C NMR chemical shifts between 172-175 ppm [9] and are additionally super-imposed. No other thylakoid components are expected to have resonance signals in this region, inferring that the pronounced peak centered at 174.2 ppm that is visible both in spectra of whole cells and of thylakoid membranes, represents the superimposed peak of accumulated lipid CO signals. This is further supported by the fact that the carbonyl signal of the most abundant lipids, MGDG and DGDG, are found here (Fig. S9). Over time, the DP spectra of whole cells show a decrease of the lipid peak at 174.2 ppm and rise of a band at 178.5 ppm, which can be attributed to carboxylic acid carbons. We attribute the rising carboxyl signal to the accumulation of FFA, supported by the HPTLC analysis in Fig. S8. Note that the intensity of protein CO band, visible in the CP-based spectra, does not change over time (Fig. S13-S16).

TAG

According to literature, the fatty acid carbonyl ^{13}C chemical shift signals of DAG fall in the region 173.1-173.4 ppm and the carbonyl signals of TAG fall in the region 172.4-172.8 ppm [9]. The DP difference spectra presented in Fig. S11B show the emergence of two small signals at ~172.9 and ~172.4 ppm after 24 h. The thylakoid spectrum does not show any structure there (Fig. S12), i.e. the signals are not from the most abundant lipid types, and they do not overlap with signals of cell-wall components, according to the comparison of wildtype and cell-wall deficient cells (Fig. S11A, blue and red). Supported by the HPTLC analysis shown in Fig.S8, we tentatively assign the emerging small bands to formation of TAG.

2. Supplementary figures

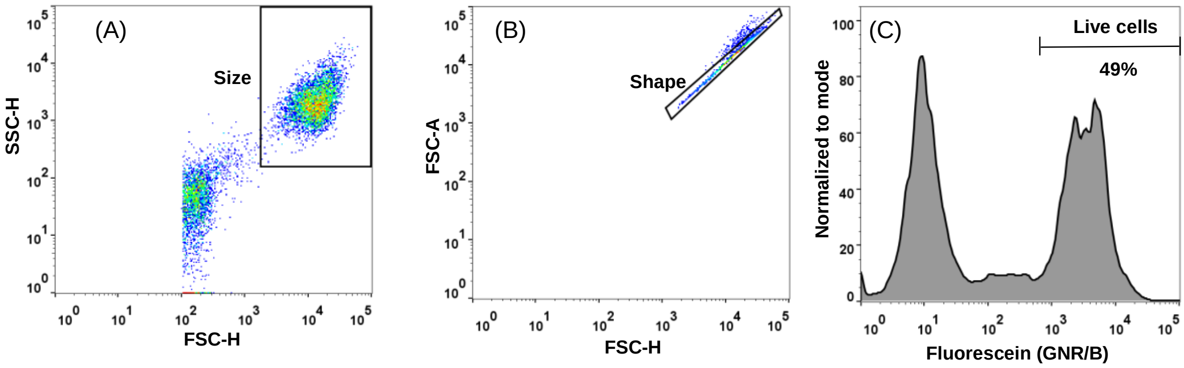

Figure S1. Representative flow cytometry plots showing the gating strategy for the *Cr.* cells stained with FDA after 3.5 hrs MAS NMR at 23 ˚C. (A) Dot plot of side scatter (SSC) vs forward scatter (FSC) was gated on cell size to select for single *Cr.* cells. (B) Dot plot of FSC-A vs FSC-H was gated on cell shape to select for single *Cr.* cells. (C) Histogram of fluorescein intensity showing a strict threshold to distinguish live (fluorescein-positive) cells from dead or unstained (fluorescein-negative) cells. Viability was quantified based on the indicated threshold for fluorescein-positive cells, and the same gating and threshold were used for all other samples.

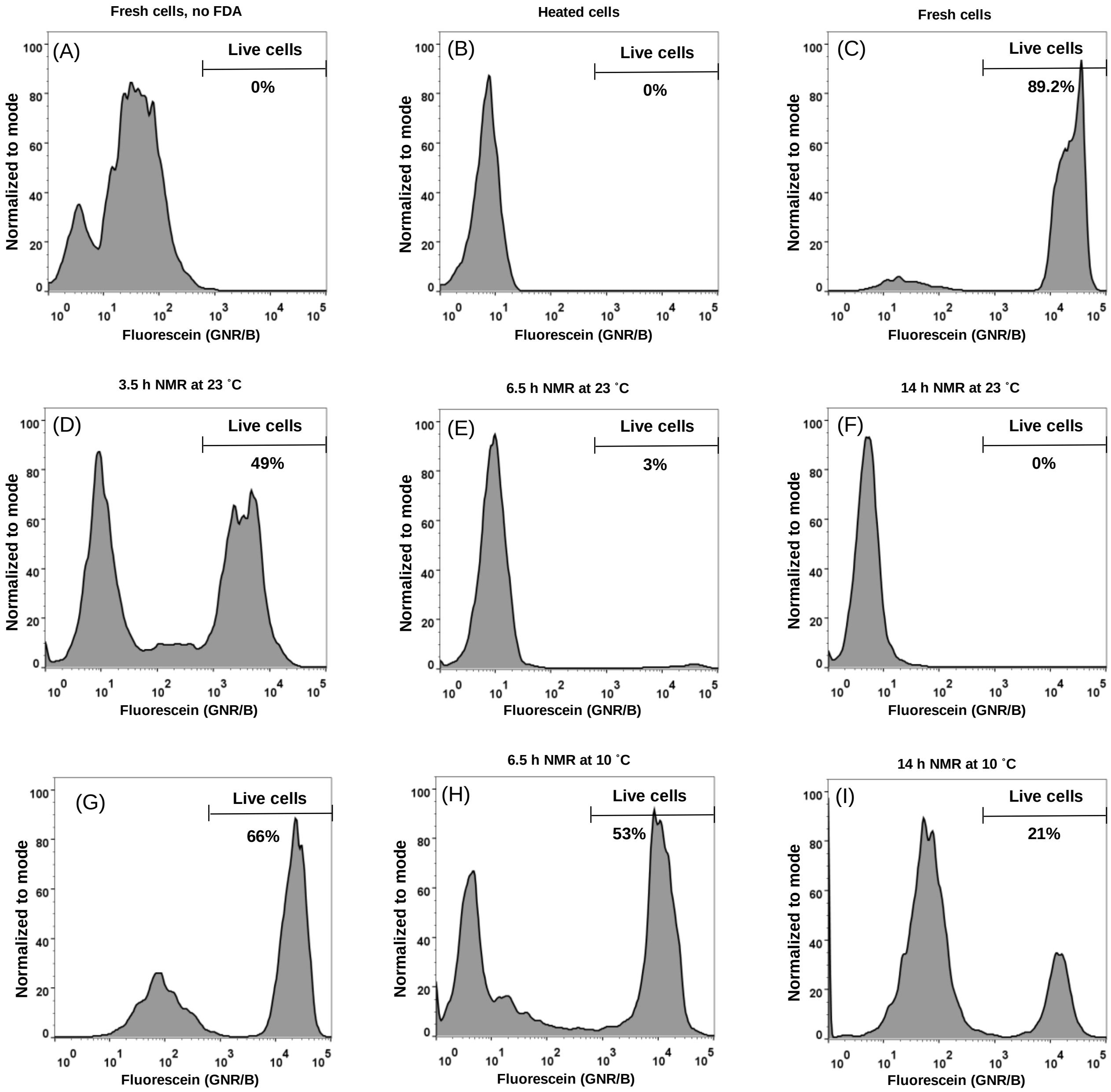

Figure S2. Representative flow cytometry histograms indicating the shift in fluorescein intensity of *Cr.* cells stained with FDA, as a readout for cell viability. (A) First negative control showing the background fluorescence of fresh unstained *Cr.* cells. (B) Second negative control showing the background fluorescence of heat-killed *Cr.* cells after FDA staining. (C) Positive control showing the fluorescence intensity of fresh *Cr.* cells after FDA staining. (D) Viability of *Cr.* cells after 3.5 h MAS ssNMR at 5 kHz spinning at 23°C and subsequent FDA staining. (E) Viability of *Cr.* cells after 6.5 h NMR at 23°C and subsequent FDA staining. (F) Viability of *Cr.* cells after 14 h NMR at 23°C and subsequent FDA staining. (G) Viability of *Cr.* cells after 3.5 h NMR at 10°C and subsequent FDA staining. (H) Viability of *Cr.* cells after 6.5 h NMR at 10°C and subsequent FDA staining. (I) Viability of *Cr.* cells after 14 h NMR at 10°C and subsequent FDA staining.

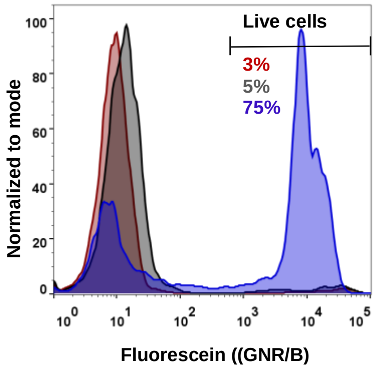

Figure S3. Flow cytometry histograms comparing fluorescein intensity of *Cr.* cells stained with FDA after 6.5 h of MAS ssNMR (red) at 23 ˚C and incubated in an 4mm MAS NMR rotor under the same cell density ($OD_{750} \approx 120$), gray) and 7 times diluted in high salt medium (blue) kept outside the magnet. The cell viability of each sample is indicated with the same colour code.

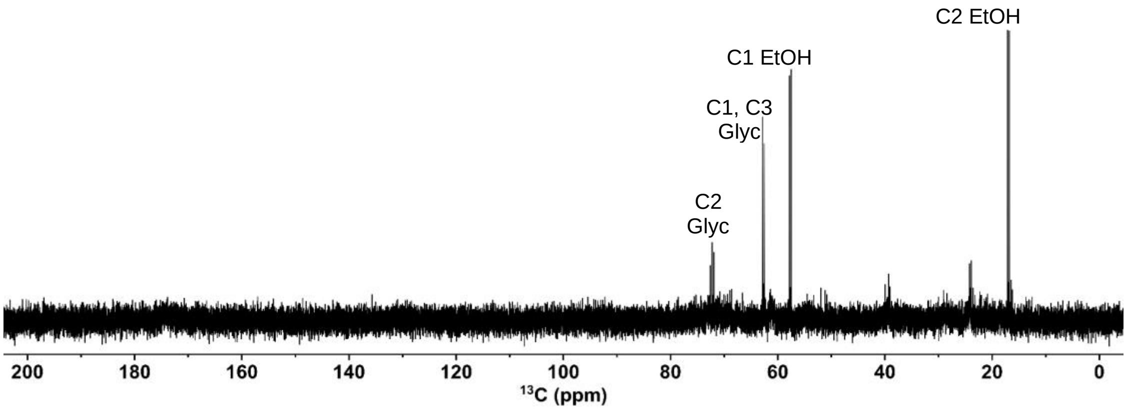

Figure S4. Proton-decoupled ^{13}C solution NMR spectrum of washed-out cells supernatant after 3.5 hours MAS ssNMR at 23 ºC. Signals of ethanol (EtOH) and glycerol (Glyc), which exerted from the cells, are labelled.

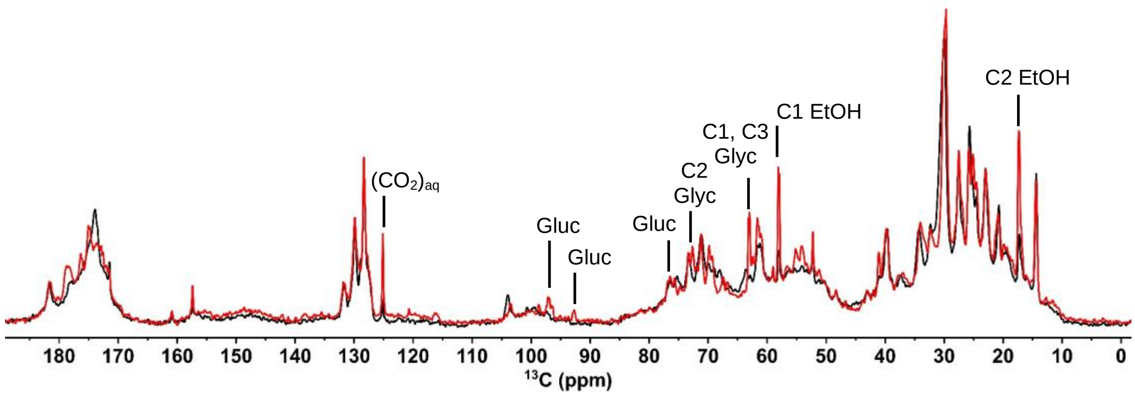

Figure S5. Overlaid DP ^{13}C ssNMR spectra of *Cr.* cells after 1 h (black) and 24 h (red) of MAS at 10 ˚C. Signals of ethanol (EtOH), glycerol (Glyc), glucose (Gluc) and aqueous CO_2 are indicated.

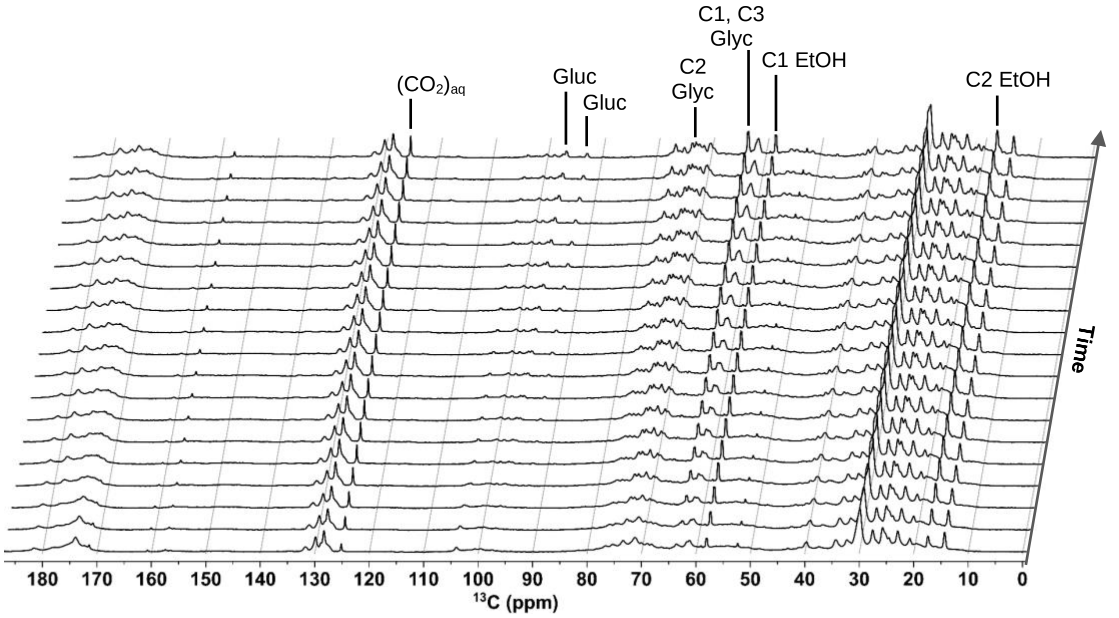

Figure S6. Stacked time-dependent DP ^{13}C ssNMR spectra of *Cr.* cells at 23 ˚C, followed for 24 hours with time intervals of 77 minutes. Signals of ethanol (EtOH), glycerol (Glyc), glucose (Gluc) and aqueous CO_2 are indicated.

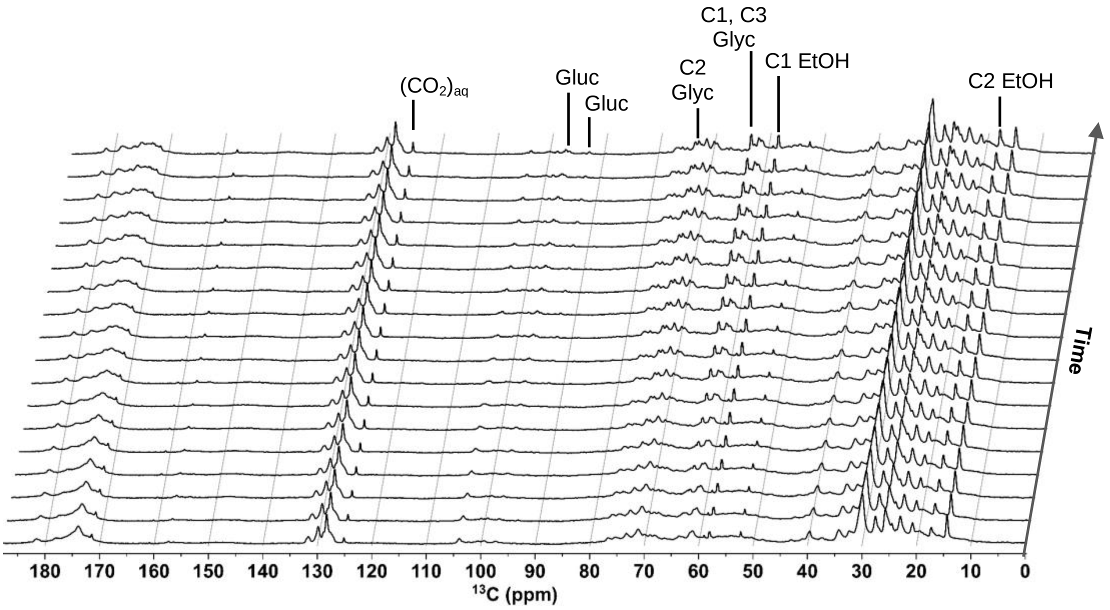

Figure S7. Stacked time-dependent DP ^{13}C ssNMR spectra of *Cr.* cells at 10 ˚C, followed for 24 hours with time intervals of 77 minutes. Signals of ethanol (EtOH), glycerol (Glyc), glucose (Gluc) and aqueous CO_2 are indicated.

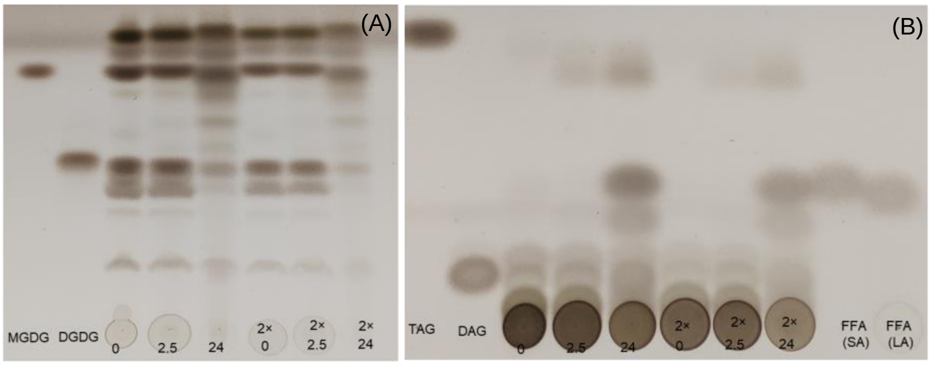

Figure S8. HPTLC analysis of total lipids content of *Cr.* cells incubated under high-cell cell density (OD_{750} ≈ 120), dark and anoxia condition at 23 ˚C and collected at different time points (0, 2.5 and 24 h). Lipids were stained using 8% (w/v) H_3PO_4 containing 10% (w/v) copper (II) sulfate pentahydrate and heated at 180 ˚C for 15min. A – Separation achieved with $CHCl_3$/MeOH/$H_2$0 (65:35:2, v/v/v) as mobile phase. B - Elution under solvent system Hexane/diethyl ether/acetic acid (70:30:1, v/v/v). Lipid identification was achieved by co-elution with the following lipid standards: MGDG - monogalactosyldiacylglycerol, DGDG - digalactosyldiacylglycerol, TAG – Oleic triglyceride, DAG – Dioleoylglycerol, FFA (SA) – stearic acid, saturated, FFA (LA) – Linoleic acid, unsaturated.

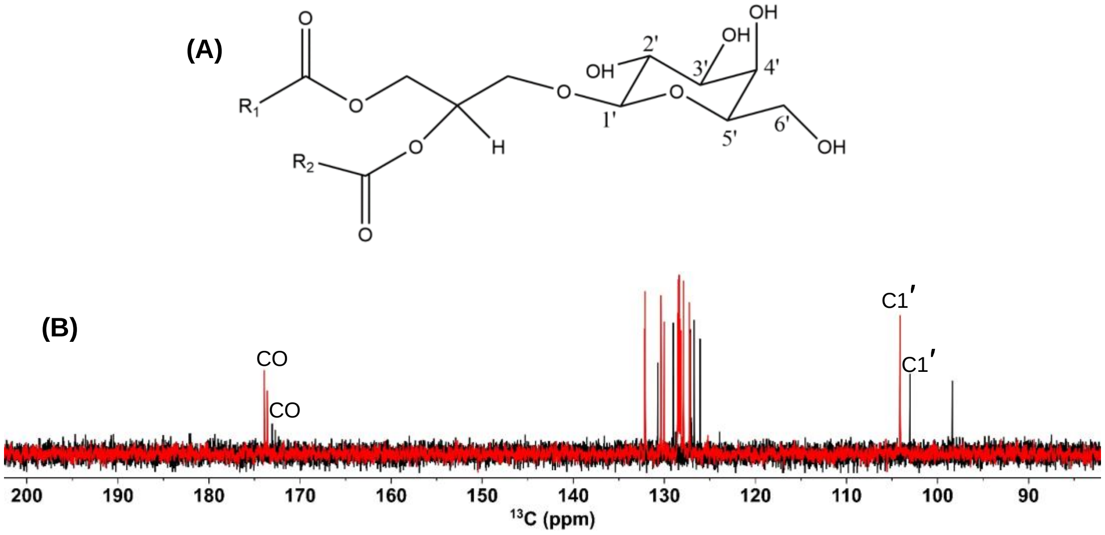

Figure S9. A. The molecular structure of MGDG. B. Proton-decoupled ^{13}C solution NMR spectra of monogalactosyldiacylglycerol (MGDG) in red and digalactosyldiacylglycerol (DGDG) in black in $CDCl_3$. The C1′ carbon of MGDG and DGDG galactosyl headgroups and the carbonyl signal (CO) are indicated.

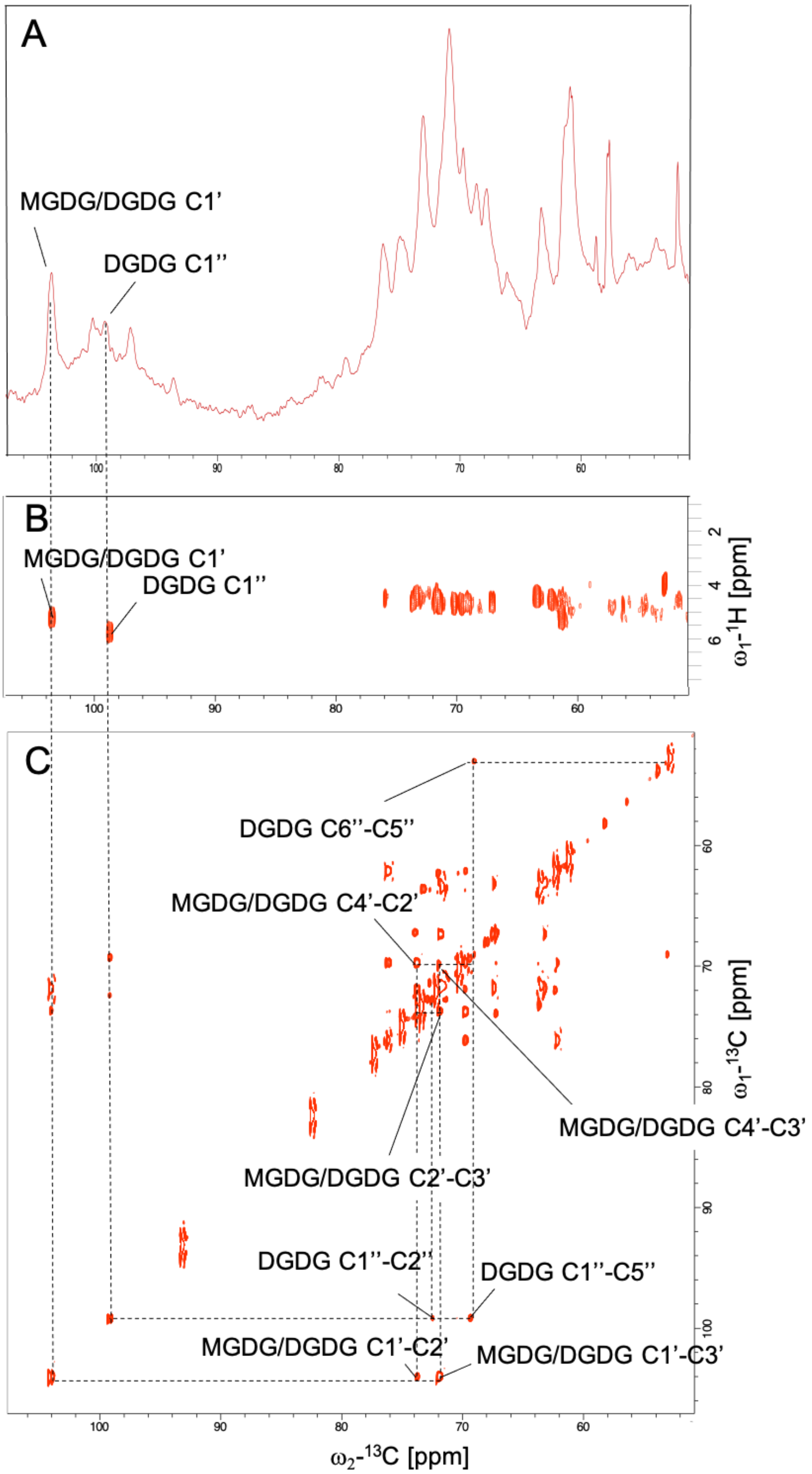

Figure S10. ^{13}C NMR assignment of the C1' carbons of MGDG or DGDG and C1'' of DGDG with help of spectra of isolated thylakoid membranes. A. DP MAS NMR spectrum of *Cr.* cells at t = 24 hrs. B. ^{1}H-^{13}C Hetcor experiment on *Cr.* isolated thylakoids. C. 13C-13C INEPT-TOBSY experiment on *Cr.* isolated thylakoids.

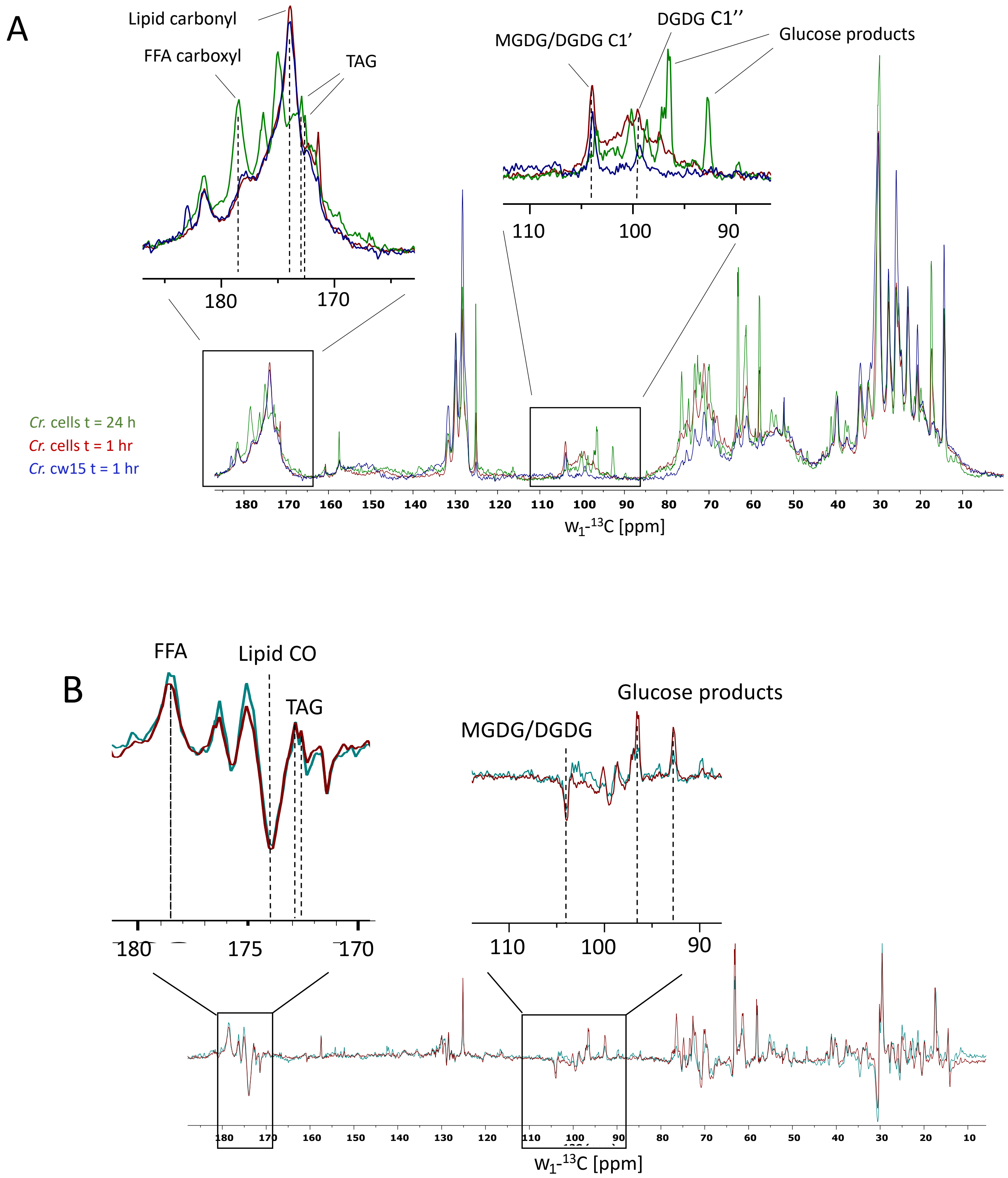

Figure S11. A: Overlaid DP ^{13}C ssNMR spectra of cell wall-deficient *Cr.* cells (cw15, blue), *Cr.* cells after 1 h NMR experiment (red) and *Cr.* cells after 24 h NMR experiment (green). The accumulated signals of lipid carbonyls, of FFA carboxyl and tentative assignments of DAG and TAG signals are indicated in the left zoomed-in spectrum. The merged signal of C1′ carbon of MGDG and DGDG galactosyl headgroups and of the DGDG C1'' carbon are indicated in the right zoomed-in spectrum. Glucose products formed after 24 h are also indicated. B: ^{13}C DP MAS difference spectrum of 24h minus 1h experiment (23 °C) for two sets of experiments (cyan and red). The insets show increase of signals attributed to FFA and TAG and decrease of lipid (left zoom-in) and increase of glucose products and decrease of galactolipid (right zoom-in).

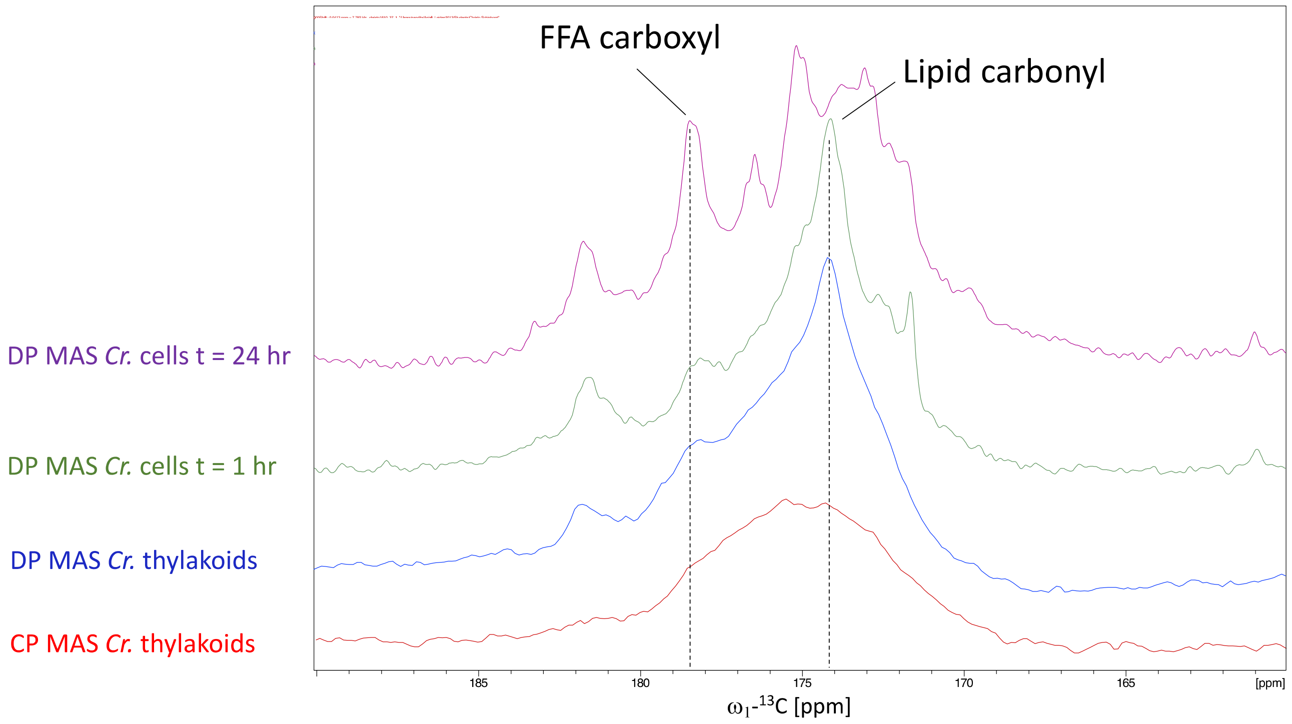

Figure S12. DP ^{13}C ssNMR spectra of *Cr.* cells after 1 hr (green) and after 24 hrs (purple) and DP (blue) and CP (red) ^{13}C ssNMR spectra of *Cr.* thylakoids. The accumulated signals of lipid carbonyls in whole cells and in thylakoids and the emerging peak of FFA carboxyl atoms are indicated.

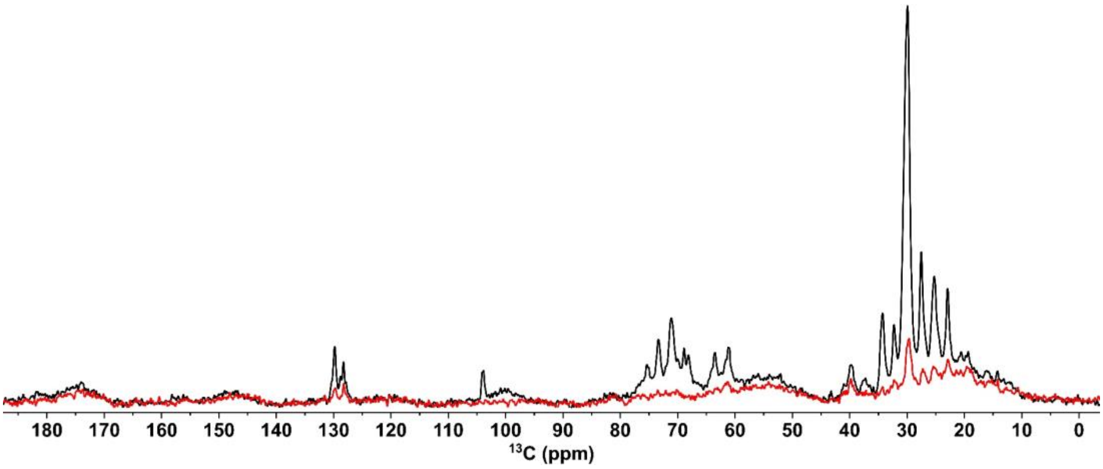

Figure S13. Overlaid CP ^{13}C ssNMR spectra of *Cr.* cells after 1 h (black) and 24 h (red) of MAS at 23 ˚C.

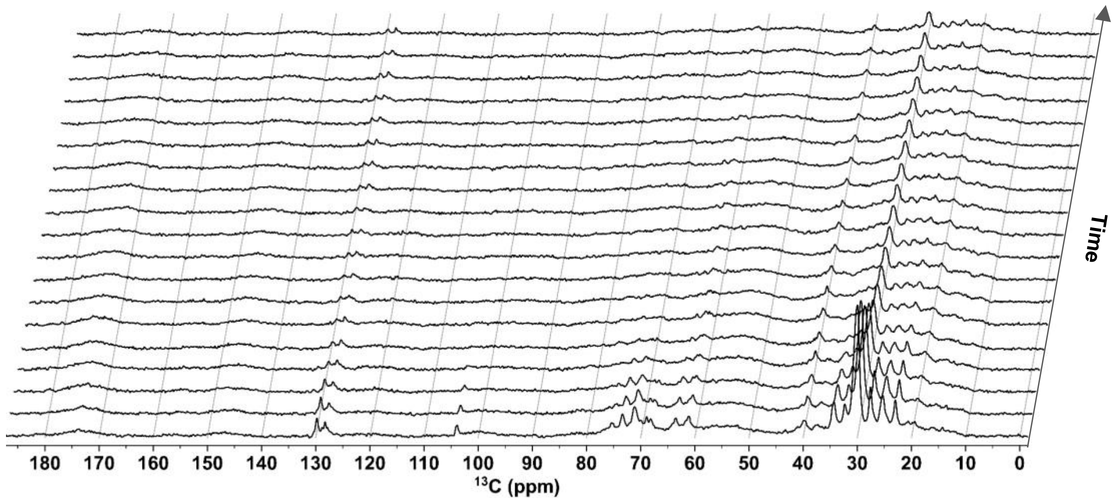

Figure S14. Stacked time-dependent CP ^{13}C ssNMR spectra of *Cr.* cells at 23 ˚C, followed for 24 hours with time intervals of 77 minutes

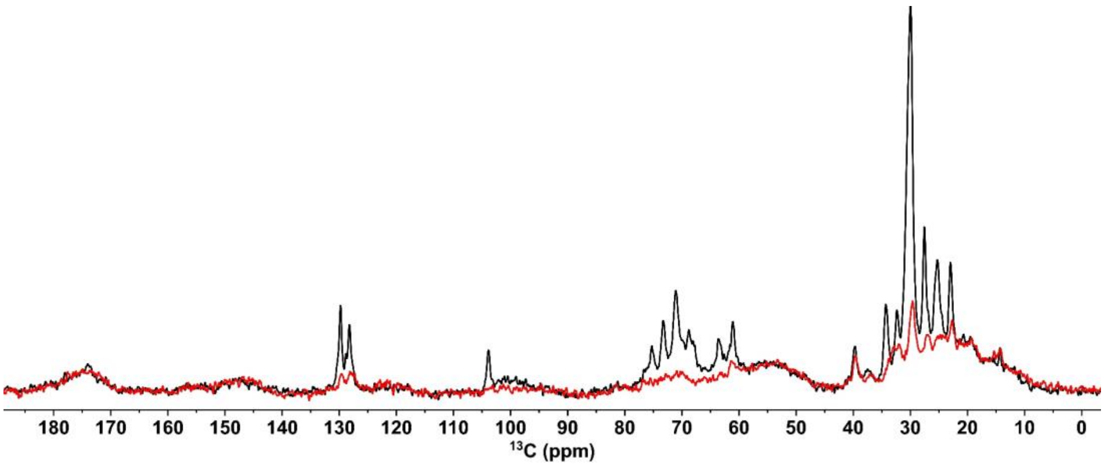

Figure S15. Overlaid CP ^{13}C ssNMR spectra of *Cr.* cells after 1 h (black) and 24 h (red) of MAS at 10 ˚C.

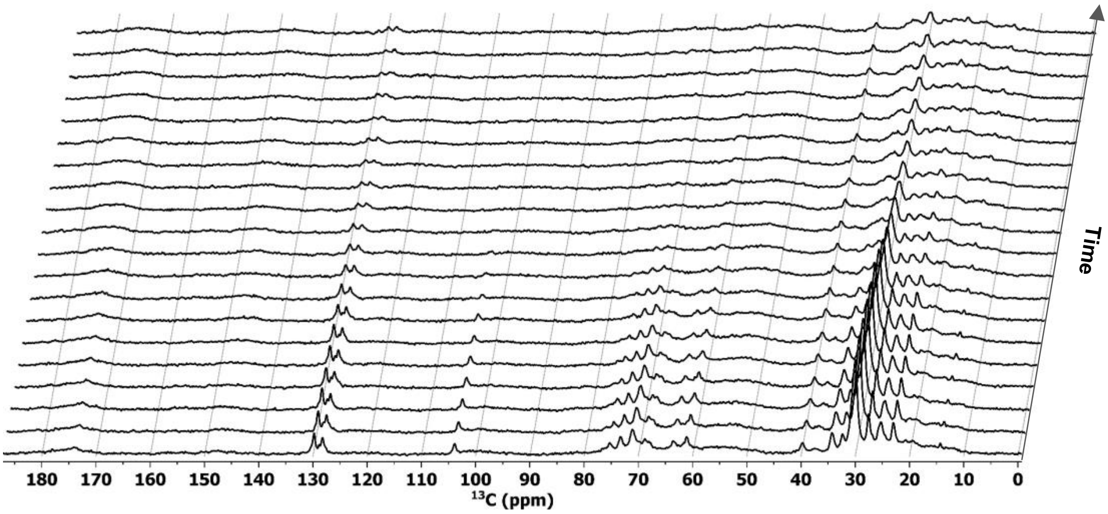

Figure S16. Stacked time-dependent CP ^{13}C ssNMR spectra of *Cr.* cells at 10 ˚C, followed for 24 hours with time intervals of 77 minutes.

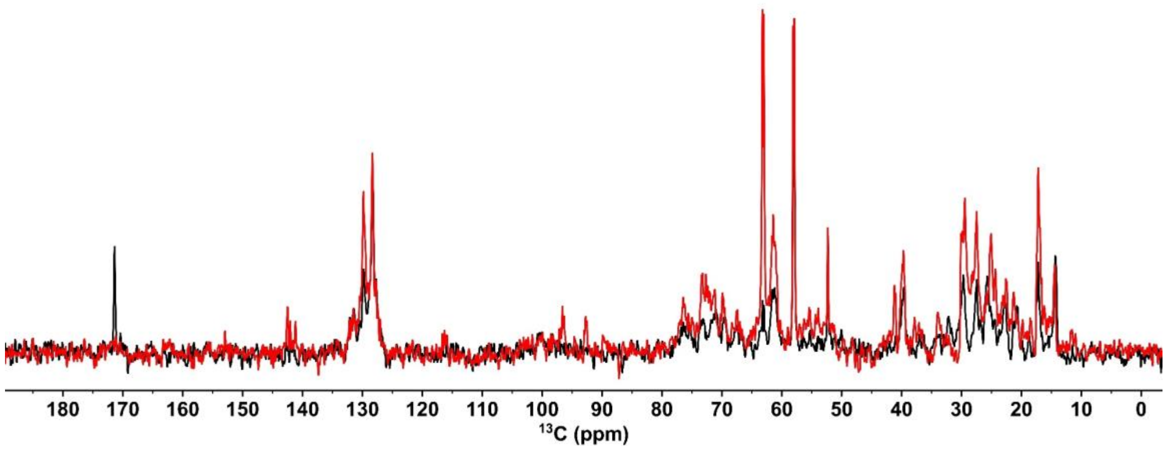

Figure S17. Overlaid INEPT ^{13}C ssNMR spectra of *Cr.* cells after 1 h (black) and 24 h (red) of MAS at 23 ˚C.

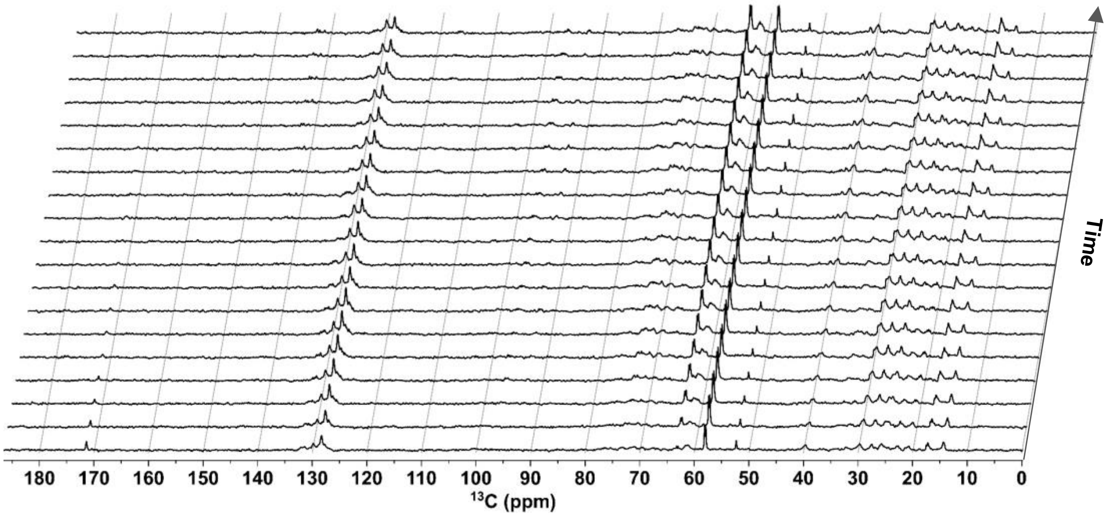

Figure S18. Stacked time-dependent INEPT ^{13}C ssNMR spectra of *Cr.* cells at 23 ˚C, followed for 24 hours with time intervals of 77 minutes.

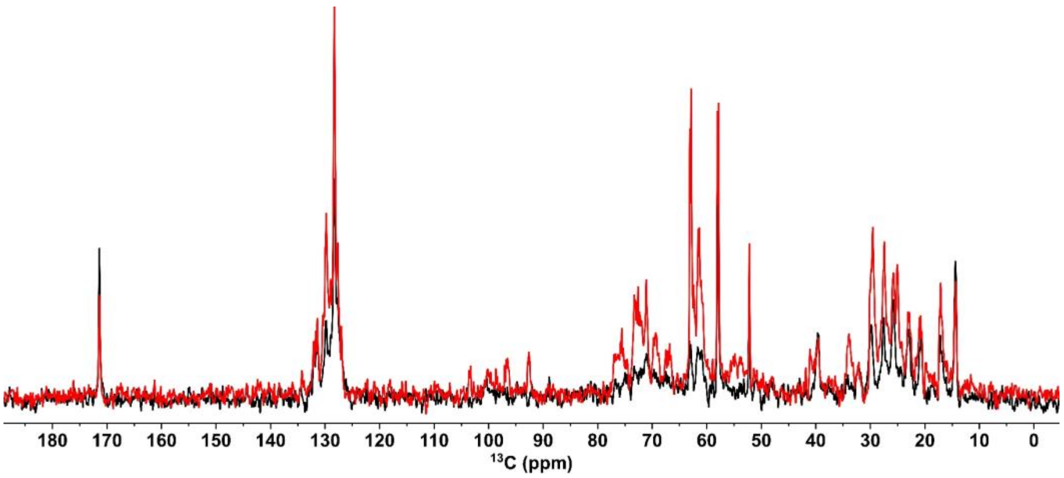

Figure S19. Overlaid INEPT ^{13}C ssNMR spectra of *Cr.* cells after 1 h (black) and 24 h (red) of MAS at 10 ˚C.

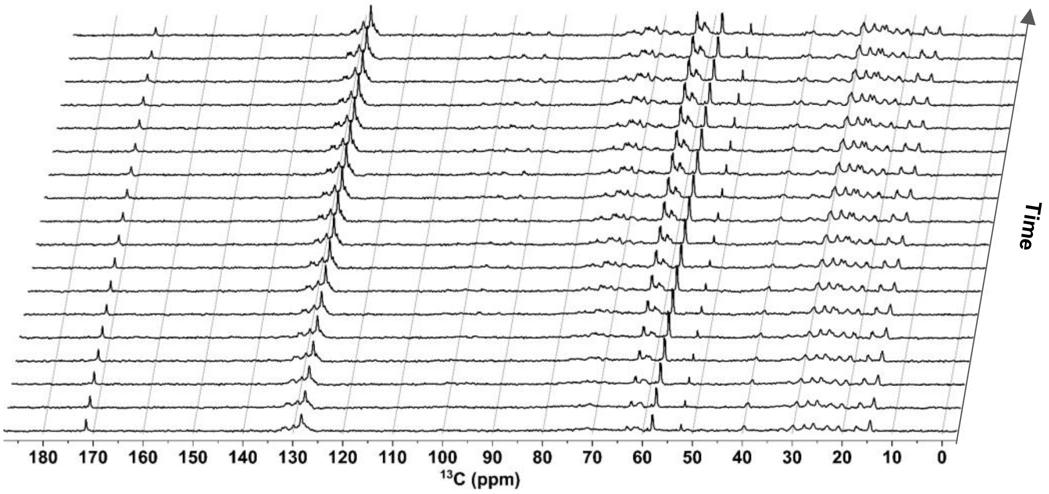

Figure S20. Stacked time-dependent INEPT ^{13}C ssNMR spectra of *Cr.* cells at 10 ˚C, followed for 24 hours with ttime intervals of 77 minutes.

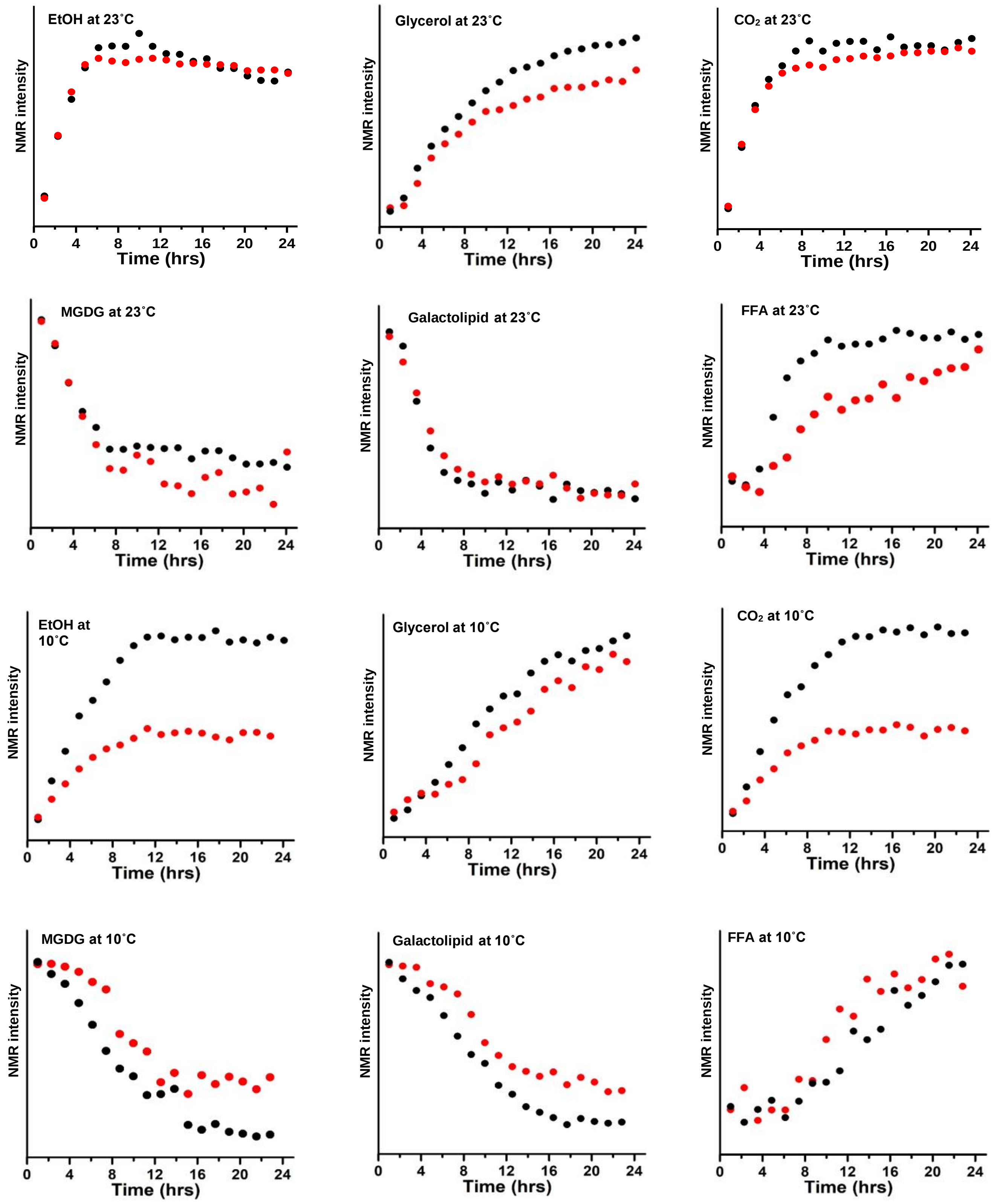

Figure S21. Reproducibility of DP NMR signal intensities for two series of experiments at 23 ˚C and 10 ˚C as a function of time for ethanol (58.1 ppm), glycerol (63.2 ppm), CO_2 (125.1 ppm) and MGDG (104 ppm), galactolipid carbonyl (174 ppm) and FFA (178.5 ppm). For visual comparison of the kinetic profiles, the intensities of spectra collected at each series are normalized at the first time point.

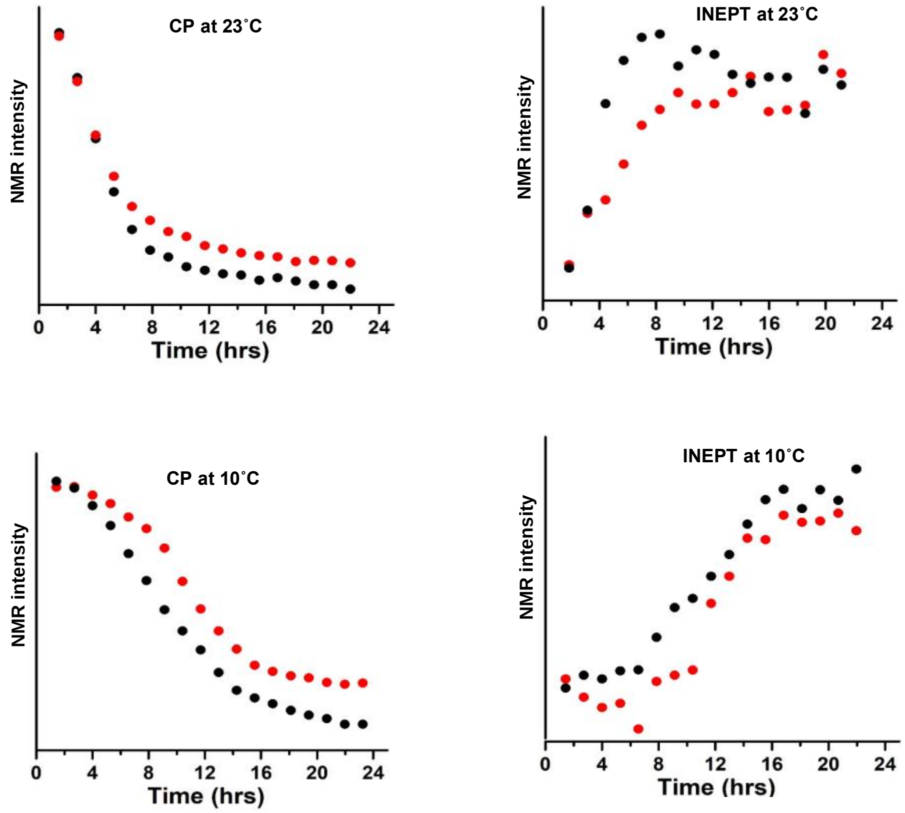

Figure S22. Reproducibility of CP and INEPT NMR signal intensities at 30 ppm for two series of experiments at 23 ˚C and 10 ˚C as a function of time. For visual comparison of the kinetic profiles, the intensities of spectra collected at each series are normalized at the first time point.

3. Supplementary table

Table ST1. Rate constants of products formed by *Cr.* upon dark concentration at high cell densities.

Product	Series 1<br>k (h^{-1}) [a]<br>23 ˚C	Series 2<br>k (h^{-1})<br>23 ˚C	Series 1<br>k (h^{-1})<br>10 ˚C	Series 2<br>k (h^{-1})<br>10 ˚C
Ethanol	0.60 ± 0.13	0.63 ± 0.065	0.25 ± 0.023	0.23 ± 0.017
Glycerol	0.12 ± 0.011	0.12 ± 0.0048	0.067 ± 0.011	0.015 ± 0.014
CO2	0.40 ± 0.022	0.38 ± 0.017	0.23 ± 0.022	0.20 ± 0.012

[a] The rate constants are obtained from single exponential fitting of NMR intensities plot versus time after concentration of the *Cr.* cells into NMR rotors.

4. References

[1] N. Sueoka, *Proc. Natl. Acad. Sci.* **1960**, *46*, 83–91.
[2] W. J. Bligh, E.G. and Dyer, *Can. J. Biochem. Physiol.* **1959**, *37*.
[3] A. Seyer, M. Cantiello, J. Bertrand-Michel, V. Roques, M. Nauze, V. Bézirard, X. Collet, D. Touboul, A. Brunelle, C. Coméra, *PLoS One* **2013**, *8*, DOI 10.1371/journal.pone.0058224.
[4] K. Yoon, D. Han, Y. Li, M. Sommerfeld, Q. Hu, *Plant Cell* **2012**, *24*, 3708–3724.
[5] A. Vieler, C. Wilhelm, R. Goss, R. Suss, J. Schiller, *Chemistry and Physics of Lipids* **2007**, 150, 143-155
[6] F. Azadi-Chegeni, M.E. Ward, G. Perin, D. Simionato, T. Morosinotto, M. Baldus, A.Pandit, *Biophys J* **2021**, 20, 270-283
[7] H. Kirchhoff, S. Haverkamp, J.F. Allen, D.B.A. Epstein, C. Mullinaux, *Plant Physiol*. **2008**, 146, 1571-1578
[8] E.L. Ulrich, H. Akutsu,J.F. Doreleijer, Y. Harano,Y.E. Ioannidis, J. Lin, M. Livny, S. Mading, D. Maziuk, Z. Miller, E. Nakatani, C. F. Schulte, D. E. Tolmie, R. K.Wenger, H. Yao, J. L. Markley, "BioMagResBank", *Nucleic Acids Research* **2008**, 36, D402-D408
[9] E.Alexandri, R. Ahmed, H. Siddiqui, M.I. Choudhary, C.G. Tsiafoulis, I.P. Gerothanassis, *Molecules* **2017**, 22, 1663

Author Contributions

((Please specify the contributions of each author including the type (e.g. data curation, funding acquisition, formal analysis, investigation, project administration, validation, writing of original draft) and the degree (e.g. lead, equal, supporting) of contribution.))

AP and FN designed the project and JA contributed to the design of the HPTLC experiments. FN performed the cell culture, NMR and flow cytometry experiments, FN and MF carried out the HPTLC experiments and TB assisted with flow cytomery measurements and analysis. FN and AP wrote the original manuscript. All authors provided critical feedback on the data analysis and contributed to the writing of the final manuscript. FN was financially supported by a Fundamental Research on Matter (FOM) Projectruimte grant of the Netherlands Organization of Scientific Research (NWO) under grant nr. 680.91.15.19. AP was financially supported by a CW-VIDI grant of the Netherlands Organization of Scientific Research (NWO) under grant nr. 723.012.103.
